# Diagnostic Issues of Asymptomatic Neurosyphilis in HIV-Positive Patients: A Retrospective Study

**DOI:** 10.3390/brainsci9100278

**Published:** 2019-10-17

**Authors:** Giancarlo Ceccarelli, Cristian Borrazzo, Alessandro Lazzaro, Giuseppe Pietro Innocenti, Luigi Celani, Eugenio Nelson Cavallari, Claudia Pinacchio, Letizia Santinelli, Claudio Maria Mastroianni, Gabriella d’Ettorre

**Affiliations:** 1 Department of Public Health and Infectious Diseases, Azienda Ospedaliero-Universitaria Policlinico Umberto I, Sapienza University, 00161 Rome, Italy; cristian.borrazzo@uniroma1.it (C.B.); alessandro1lazzaro@gmail.com (A.L.); giuseppepietro.innocenti@uniroma1.it (G.P.I.); luigi.celani@uniroma1.it (L.C.); eugenionelson.cavallari@uniroma1.it (E.N.C.); claudia.pinacchio@uniroma1.it (C.P.); letizia.santinelli@uniroma1.it (L.S.); claudio.mastroianni@uniroma1.it (C.M.M.); gabriella.dettorre@uniroma1.it (G.d.); 2 Department of Medical Sciences, Infectious Diseases, University of Turin, 10124 Turin, Italy

**Keywords:** sexual health, HIV, antiretroviral therapy, CD4, syphilis, neurosyphilis

## Abstract

**Introduction**: Asymptomatic neurosyphilis (ANS) is a disease that is difficult to diagnose in people living with HIV (PLWH). The European Guidelines on the management of syphilis suggest that ANS should be suspected and thus the lumbar puncture (LP) should be performed in cases of (1) late syphilis (acquired >2 years previously), (2) CD4^+^ cells ≤ 350/mm^3^ and/or a serum Venereal Disease Research Laboratory/Rapid Plasma Reagin (VDRL/RPR) title > 1:32, (3) “serological failure” after syphilis therapy, and (4) the use of alternative treatment for syphilis. In the present study, we aimed to verify the accuracy of the guideline’s criteria for the indication of LP in the suspicion of ANS in a cohort of PLWH. **Methods**: This retrospective study was carried out in a cohort of PLWH referred at a single medical center of a large academic hospital in Italy. Clinical and laboratory data of patients diagnosed with late syphilis were extracted from the cohort and analyzed. The European Guidelines of syphilis were adopted for patient management. **Results**: Out of a cohort of 713 PLWH, only 51 (7%) had a diagnosis of late syphilis and were therefore included in the study. Thirty-one subjects (61%) met one or more diagnostic criteria to perform LP: 39% (12/31) of patients undergoing LP had a diagnosis of ANS. The accuracy of predictive criteria for ANS, suggested by the guidelines, was 62% for RPR > 1:32 and 74% for CD4^+^ ≤ 350 cc/µL. The simultaneous occurrence of both criteria (RPR > 1:32 plus CD4^+^ ≤ 350 cc/µL) achieved a diagnostic accuracy of 59%. Interestingly, only 17% of patients who underwent LP for serological failure were eventually diagnosed positive for ANS. **Conclusion**: Asymptomatic neurosyphilis represents a challenging, but not uncommon, diagnosis. Therefore, it requires a careful investigation. Low CD4^+^ cell count and RPR > 1:32 remain excellent predictors of neurosyphilis, but have become the only acceptable predictors of ANS in PLWH. “Serologic failure” should be regarded with caution as a criterion to perform LP in order to investigate possible ANS in HIV-syphilis coinfected patients asymptomatic for neurological disorders. The retrospective nature of this single-site study may represent a limit to the interpretation of the data. Thus, larger clinical studies on the topic are warranted.

## 1. Introduction

Neurosyphilis (NS) is the infection of the central nervous system (CNS) by *Treponema pallidum*, subspecies pallidum (TP), and can occur at any time after initial infection independently of human immunodeficiency virus (HIV) coinfection. Taylor et al. reported a 2.1% incidence of NS among people living with HIV (PLWH) compared to 0.6% among people without HIV, whereas Lynn and Lightman found in 2004 that 23.5% of untreated patients with HIV-coinfection had NS compared to only 10% of untreated patients without HIV [1]. This increased rate of early NS among PLWH may be related to the immunological system impairment rather than the CNS invasion [2], considering that patients with CD4 < 350 cell/mm^3^ have a three-fold increase in neurological involvement [3]. 

The diagnosis of NS is usually based on neurological impairment and confirmed by examination of the cerebrospinal fluid (CSF) through lumbar puncture (LP). For these reasons, CSF assessment is mainly indicated in patients with neurological signs or symptoms (also ocular or auricular) regardless of the stage of the disease or in the event of tertiary syphilis [4,5,6]. On the other hand, the diagnosis of so-called “asymptomatic” neurosyphilis (ASN) is more challenging and controversial, especially in case of coinfection with HIV. The European Guidelines of syphilis underlined that most of current definitions rely on a combination of CSF laboratory tests (protein, cells, CSF TT, and CSF NTT) in patients asymptomatic for neurological signs or symptoms, but no consensual definition exists.

To manage these hard-to-diagnose cases, the guidelines suggest that LP should be performed in all PLWH asymptomatic for neurological signs/symptoms with late syphilis and CD4^+^ cells ≤ 350/mm^3^ and/or a serum Venereal Disease Research Laboratory/Rapid Plasma Reagin (VDRL/RPR) title > 1:32 in the case of “serological failure” after syphilis therapy or the use of alternative treatment during late syphilis [7]. However, LP remain an invasive procedure with several potential complications (i.e., spinal hematoma, epidermoid tumor implantation, uncal or transtentorial herniation, neurological deterioration seeding of infection to the CSF). For this reason, it should be performed only when benefits outweigh the risks.

On the grounds of these assumptions, we carried out a retrospective study to verify the accuracy of the criteria for LP suggested by the European Guidelines in a cohort of PLWH with suspected ANS [7].

## 2. Methods

### 2.1. Enrollment

This retrospective study was carried out in a cohort of PLWH referred at the infectious disease outpatient clinic of a large academic hospital sited in central Italy. Clinical and laboratory data of patients with a diagnosis of late syphilis were extracted from the medical records of this cohort and analyzed. Patients under the age of 18 or patients affected by neurological diseases or by causes of possible false-positive nontreponemal and treponemal tests were not included in the analysis. 

### 2.2. Clinical Data Collection

Demographics, medical history (coinfections, co-pathologies, and antiretroviral treatments (ART)) and clinical data at the time of syphilis diagnosis were collected from existing medical records. Laboratory data reported in clinical files included: (i) Results of screening tests for syphilis (IgM, RPR, and TPHA), (ii) immuno-virologic status at the time of HIV and syphilis diagnosis (lymphocyte subpopulations in peripheral blood (CD4^+^ and CD8^+^), and HIV-RNA (measured with Versant kPCR—Siemens Healthcare Diagnostic Inc., Tarrytown, NY, USA) with a detection limit of 37 copies/mL). 

### 2.3. Guidelines, Definitions, and Criteria Used in the Management of the Cohort of PLWH

Criteria adopted for the management of all patients followed at the clinic complied with the European Guidelines for syphilis and the Italian Guidelines for HIV. According with European Guidelines for syphilis, LP was performed in PLWH, asymptomatic for neurological signs or symptoms, with diagnosis of late syphilis and CD4^+^ cell count ≤ 350/mm^3^ and/or a serum VDRL/RPR title > 1:32, in the case of serological failure or the use of alternative treatment [7]. In subjects matching the criteria for ANS, CSF analysis was performed (CSF-RPR, CSF-TPHA, CSF-white blood cells, CSF-proteins, pleocytosis, neurotropic viruses). Asymptomatic neurosyphilis was defined and diagnosed by a reactive CSF RPR test (or CSF white blood cells >20/μL plus a reactive CSF *TP* particle agglutination ≥ 1:640.) [7,8] in PLWH asymptomatic for neurological signs or symptoms. Late syphilis was defined as syphilis acquired >2 years previously according with the World Health Organization (WHO) and European Guidelines for syphilis [7]. Serological failure was defined as a lack of a four-fold decrease in RPR titers at 12 months after initial treatment.

### 2.4. Statistical Analysis 

The software used for the statistical analysis was performed using SPSS (version 22.0; SPSS Inc., Chicago, IL, USA). Most of the statistical tests were performed using paired comparisons. To assess the distribution, the Kolmogorow-Smirnov test was used. Group comparisons were made by Student’s unpaired two-tailed *t* test or Mann Whitney test as appropriate. The Chi-square tests were used to compare the differences between proportions. Spearman’s rank correlation was used to analyze the correlation between RPR and CD4^+^ reactivity. Receiver operating characteristic (ROC) analysis was performed to determine the performance of RPR and CD4^+^ in neurosyphilis and the optimal cut-off points were determined corresponding to the Youden’s index. Results were considered statistically significant when the p-value was less or equal to a threshold of 0.05. 

### 2.5. Ethical Issues

The study was approved by the institutional review board of “Sapienza” University of Rome (VCD.22/8/18). Patients included in the study signed the informed consent and the research was conducted in accordance with the Helsinki Declaration of 1975 (revised in 2000). This study was compliant with requirements of Standards for Reporting of Diagnostic Accuracy Studies (STARD) statements [9].

## 3. Results

### 3.1. Demographic and Clinical Characteristics of Patients Enrolled 

**Figure 1:**
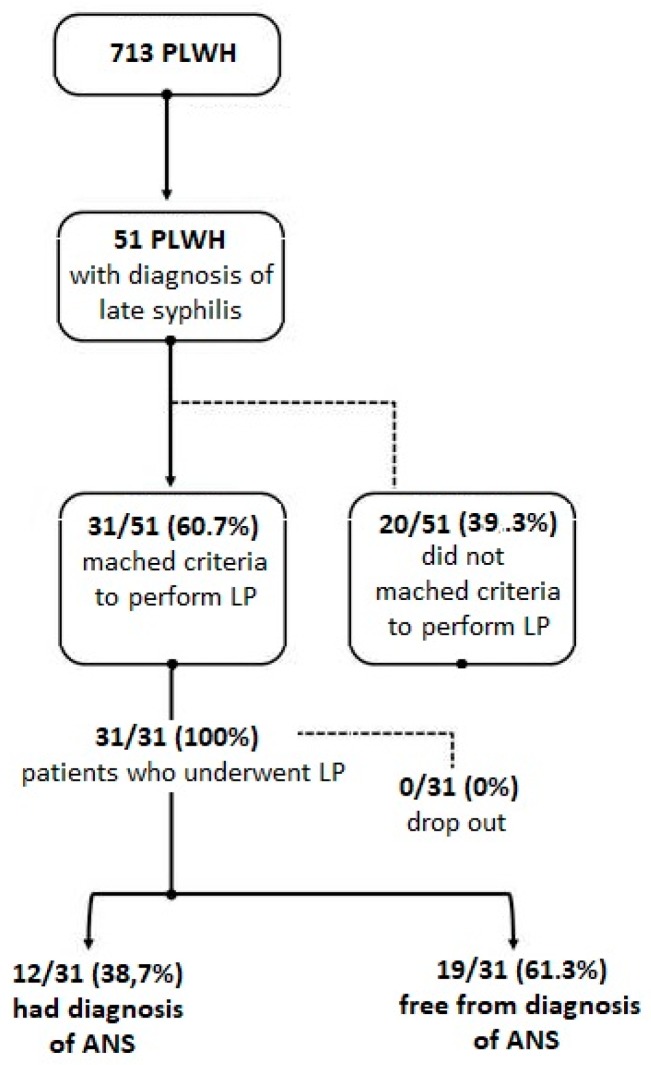
Standards for Reporting of Diagnostic Accuracy Studies (STARD) diagram to report flow of participants through the study.

Out of a cohort of 713 PLWH, only 51 (7%; 49 male and 2 female) had a diagnosis of late syphilis and were therefore included in the study (Figure 1). The median age was 46 years (IQR: 25–75%: 42–51). The median time of HIV infection was 11 years (IQR 6–18), nadir CD4^+^ (the person’s lowest CD4 count) 301 cells/mm^3^ (IQR 114.0–352.5), and actual CD4^+^ (the person’s most recent CD4 count) 522 cells/mm^3^ (IQR: 342–706.2). The count of CD4^+^ at the enrollment was significantly different from CD4^+^ nadir (*p*-value = 0.001). All patients enrolled were under prolonged and effective antiretroviral therapy and had HIV-RNA < 37 copies/mL.

### 3.2. Evaluation of ANS

Out of 51 PLWH with a diagnosis of late syphilis, 31 (61%) subjects matched one or more criteria to perform LP in accordance with European Guideline [7]. Thirty-nine percent (12/31) of patients undergoing LP had a diagnosis of ANS (Figure 1). All 12 patients diagnosed with ANS had a reactive CSF RPR test, and five subjects simultaneously presented CSF white blood cells >20/μL (median 45/μL; min 30/μL, max 61/μL). Normal CSF proteins and glucose concentration were found in all cases. The distribution of variables among our population and the statistical correlation with the diagnosis of NS are reported in Table 1. 

### 3.3. Accuracy of Diagnostic Criteria Suggested to Perform LP for Suspicion of ANS

**Figure 2:**
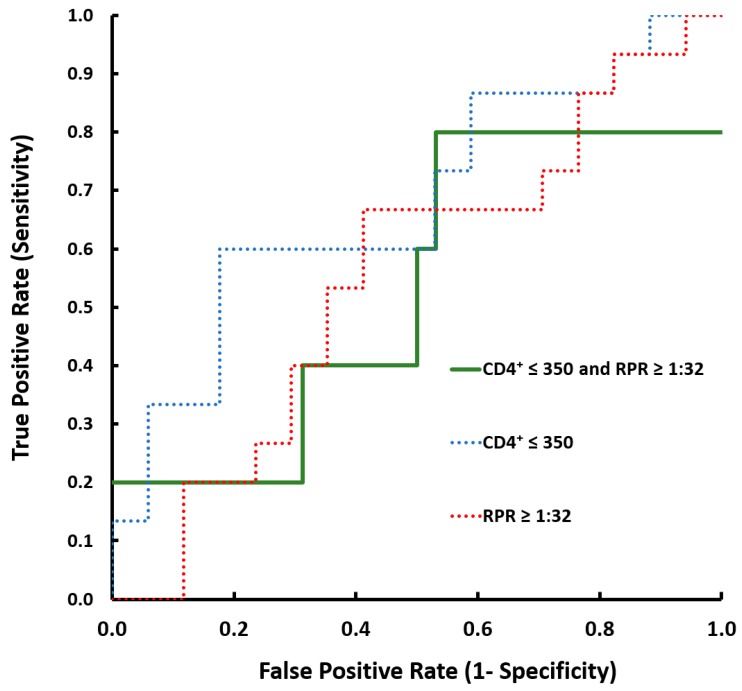
Receiver operating characteristic (ROC) curve of the CD4^+^ and/or Rapid Plasma Reagin (RPR) to detect the disease, calculated assuming different cut-offs. (CD4^+^ expressed in cells/mm^3^).

Receiver operating characteristic curve analyses were performed to identify the predictors of accuracy in the diagnosis of asymptomatic NS (Figure 2). In particular, using a threshold of CD4^+^ ≤ 350 cells/mm^3^, sensitivity was 75% and specificity 82%, with a diagnostic accuracy of 72%. With RPR threshold of ≥ 1:32, sensitivity was 67% and specificity 59%, with a diagnostic accuracy of 62%. Finally, for threshold combining CD4^+^ ≤ 350 cells/mm^3^ and RPR ≥ 1:32, sensitivity was 50% and specificity 67%, with a diagnostic accuracy of 58%. The ROC curve analysis is shown in Table 2. Regarding “serological failure”, 83% (10/12) of the patients who met this criterion and underwent LP were negative for ANS. In our cohort, no patients were treated with inappropriate or second-line therapy.

### 3.4. Correlation between Diagnosis of Asymptomatic NS and Nadir of CD4^+^

When we explored the chance to improve the performance of diagnostic tool for diagnosis of ANS proposed by the European Guidelines, we evaluated nadir of CD4 as adjunctive criterion. 

**Figure 3:**
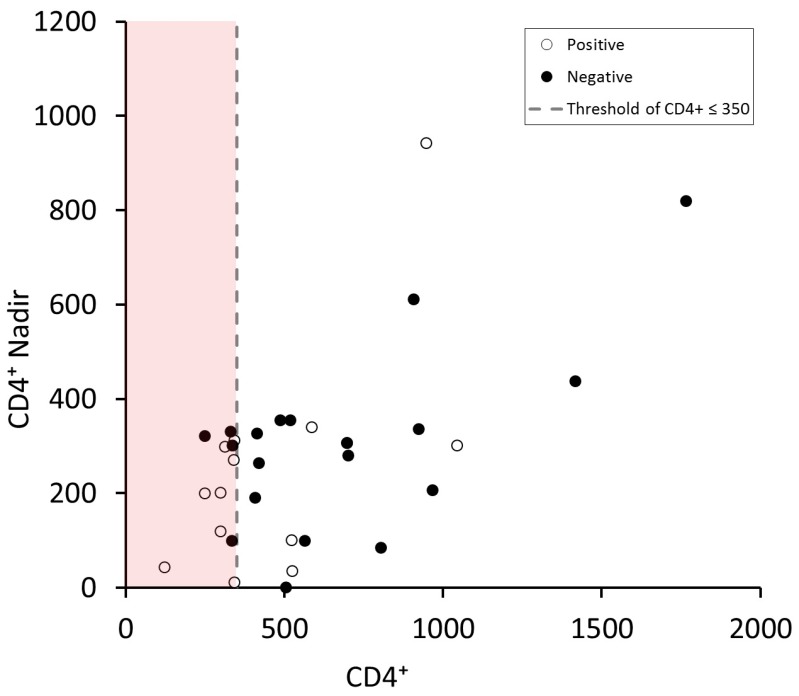
Correlation between CD4^+^ and CD4^+^ nadir white and black circles represent the negative and positive patients. (CD4 expressed in cells/mm^3^).

As shown in Figure 3, the performance of diagnostic tool was not improved using nadir of CD4^+^ as additional parameter. 

## 4. Discussion

Syphilis in PLWH may have atypical presentation and accelerated progression, and different stages of the disease may overlap [10,11,12]. The involvement of CNS can occur at any stage of disease in course of HIV infection and the clinical presentation may differ significantly, being generally characterized by more or less evident neurological symptoms, but sometimes also being entirely asymptomatic [4,5,6]. 

Currently, despite the lack of consensus on the diagnostic criteria for ANS [7], most studies agree on the generic definition of a pathology characterized by a CSF finding compatible with a neurosyphilis diagnosis in the absence of neurological signs or symptoms. For these reasons, although the definition of ANS remains difficult to clarify and its epidemiological impact has been poorly studied, the most recent guidelines on syphilis definitively recognize this nosological entity [7,8,13]. Actually, in PLWH without neurological signs but with a significant suspicious of ANS due to low levels of CD4 (≤350 cells/mm^3^) and/or VDRL/RPR title > 1:32, serological failure after previous adequate antibiotic treatment or the use of alternative drugs, the diagnosis of ANS is based principally on CSF abnormalities [13]. Since LP is an invasive procedure and is not devoid of risks of serious complications (e.g., CNS infections, cerebral herniation), assessing the effectiveness of the criteria used to identify patients at risk for neurosyphilis is crucial. 

In our cohort, among 51 participants with a diagnosis of late syphilis enrolled, 31 (60%) fulfilled criteria to undergo LP (according with European Guideline for syphilis) and only 12 (23%) were positive for ANS. In subjects undergoing LP, CSF analysis was compatible with a diagnosis of NS in 39% (12/31) of the cases, while the CSF analysis was normal in the remaining 61% (19/31). When we analyzed the low performance levels of the tool suggested by the guidelines to evaluate the opportunity to perform LP in suspected ANS, we noticed some interesting features not previously highlighted in the literature. First, we observed that the accuracy of either RPR> 1:32 or CD4^+^ ≤ 350 cells/mm^3^ as predictive criteria of ANS were of 62% and 74%, respectively, when taken into account individually. On the other hand, the criteria including both RPR> 1:32 and CD4^+^ ≤ 350 cells/mm^3^ achieved a diagnostic accuracy of 59%. Interestingly, only 17% (2/12) of patients undergoing LP for serological failure were positive for ANS. In our cohort, no patients were treated with inappropriate or second-line therapy. 

These data underline that low CD4^+^ cell counts and RPR > 1:32 remain excellent predictors of neurosyphilis and have become only acceptable predictors of ANS in PLWH, as serological failure is not entirely reliable and can be misleading. A possible explanation of this data might be that a portion of the patients classified as having “serological failure” did not really present a failure, but instead underwent reinfections not reported in the anamnesis. This hypothesis may partially explain the difficulty in resolving the infection misinterpreted as a serological failure. Moreover, the “definition” of ANS (given by the guidelines) is based on the diagnosis of neurosyphilis (obtained by the results of LP) mitigated by the clinical observation of the absence of neurological symptoms. Therefore, it is reasonable to suppose that patients with ANS represent an intermediate step between the absence and presence of neurosyphilis, or more likely, a less severe clinical manifestation of neurosyphilis. However, in the case of ANS, it should be noted that the criteria used to evaluate the opportunity to perform the LP were taken directly from the neurosyphilis diagnostic tool. These criteria, having been calibrated for the diagnosis of neurosyphilis, may not accurately assess the indication to perform LP in the case of ANS. Accordingly, it is possible that if neurosyphilis is more frequent among subjects with CD4 < 350 cells/mm^3^, this data is not as stringent for patients with ANS who suffer from a less severe condition. 

Finally, considering that a patient’s lowest ever (or nadir) CD4 cell count is a predictor of frailty severity and is strongly correlated with a number of worst outcomes that occur later in the progression of HIV disease, we explored the chance to improve the performance of the diagnostic tool proposed by the European Guideline with this parameter [14,15,16,17]. As shown in Figure 2, although implementing the sensitivity of the diagnostic tool using additional data appears a suggestive idea, the value of nadir of CD4^+^ was not suitable for this purpose according to our analysis. However, because of the small number (12/51) of subjects with ANS in our cohort, the interpretation of these findings must be approached with caution.

Accordingly, the major limitation of this study is the small sample size and the related statistical concerns. However, the low frequency of the pathology analyzed could justify these weaknesses. Moreover, this study suffers from the availability of retrospective health record data only from a single large academic medical center. For these reasons, the study should be considered as a pilot research and should prompt national or international collaboration to pool a larger dataset. 

## 5. Conclusions

Neurosyphilis continues to occur in the context of cycling syphilis outbreaks. Therefore, case identification is relevant considering the global resurgence of syphilis [18]. The results of this single-site retrospective study open a critical evaluation of the diagnostic systems currently in use, but must be evaluated carefully and cautiously [18,19]. 

Even with all the previously highlighted limits, according to our data, the “serologic failure” does not seem to perform well in predicting the diagnosis of ANS and should be used with caution as a criterion to perform LP in PLWH asymptomatic for neurological disorders.

Furthermore, our data highlight that ANS is a nosological entity that is difficult to diagnose. However, ANS is not rare, and is therefore worthy of being carefully researched. Large clinical studies are required to evaluate the chance of improving the diagnostic tool proposed by the current guidelines in order to reduce the number of unnecessary LP in the diagnosis of ANS. 

## Figures and Tables

**Table 1:** Distribution of variables among participants and their statistical association with asymptomatic neurosyphilis (NS) diagnosis assessed by the Wilcoxon-Mann-Whitney test (*p*-value < 0.05). Percentages were calculated as the number of patients with threshold significant on the whole analyzed positive and negative subjects. (CD4^+^ expressed in cells/mm^3^).

Parameters	Total (*n* = 31)		Patients with ANS (*n* = 12)		Free for Diagnosis of ANS (*n* = 19)		OR	
	No.	(%)	No.	(%)	No.	(%)	(95% CI)	*p*-Value
RPR ≥ 1:32	12/31	(39)	6/12	(50)	6/19	(32)	2.2 (0.5 to 9.6)	0.307
CD4^+^ ≤ 350	10/31	(32)	8/12	(67)	2/19	(89)	16.8 (2.5 to 70)	0.003
CD4^+^ ≤ 350 or RPR ≥ 1:32	14/31	(45)	8/12	(67)	6/19	(32)	10.8 (1.8 to 65)	0.009
CD4^+^ ≤ 350 and RPR ≥ 1:32	6/31	(19)	2/12	(17)	4/19	(21)	0.7 (0.11 to 4.9)	0.763
CD4^+^ ≤ 350 and/or RPR ≥ 1:32	20/31	(64)	10/12	(83)	10/19	(53)	4.5 (1 to 27)	0.094
Serological failure	12/31	(39)	2/12	(17)	10/18	(55)	0.2 (0.1 to 1)	0.057

**Table 2:** Comparison of the area under the ROC curve (AUC) and optimal sensitivities and specificities of each parameter are shown in the associated table. Columns show: Parameters, negative cases, positive cases, sensitivity, specificity, negative predictive value (NPV), diagnostic accuracy, and AUC. (CD4^+^ expressed in cells/mm^3^).

		Patients with ANS	Free from Diagnosis of ANS	Sensitivity		Specificity		NPV	Accuracy	AUC
Parameters	Condition	No.	No.	(%)	95%CI	(%)	95%CI	(%)	(%)	(%)
RPR ≥ 1:32	Yes	6	6	67%	48% to 83%	59%	39% to 75%	74%	62%	53%
	No	6	13							
CD4^+^ ≤ 350	Yes	8	2	75%	42% to 94%	70%	46% to 88%	82%	74%	72%
	No	4	17							
CD4^+^ ≤ 350 or RPR ≥ 1:32	Yes	8	6	83%	36% to 98%	61%	41% to 79%	94%	82%	76%
	No	4	13							
CD4^+^ ≤ 350 and RPR ≥ 1:32	Yes	2	4	50%	30% to 70%	67%	44% to 96%	87%	59%	58%
	No	10	15							
CD4^+^ ≤ 350 and/or RPR ≥ 1:32	Yes	10	10	62%	51% to 98%	83%	42% to 79%	94%	82%	73%
	No	2	9							

## Data Availability

Scientific material is available from the authors, upon request.
